# The mitochondrial genome of *Stereolepis doederleini* (Pempheriformes: Polyprionidae) and mitogenomic phylogeny of Pempheriformes

**DOI:** 10.1590/1678-4685-GMB-2020-0166

**Published:** 2021-03-03

**Authors:** Dae-Ju Oh, Jong-Chul Lee, Young-Min Ham, Yong-Hwan Jung

**Affiliations:** 1 Biodiversity Research Institute, Jeju Technopark, Seogwipo, Republic of Korea.

**Keywords:** Bayesian inference, maximum-likelihood, mitochondrion, neighbor-joining, striped jewfish

## Abstract

The complete mitochondrial (mt) genome of *Stereolepis doederleini* was sequenced from a specimen collected in a commercial aquarium in Jeju Island. The sequence was 16,513 base pairs in length and, similar to other vertebrate mt genomes, included 37 mt genes and a noncoding control region; the gene order was identical to that of typical vertebrate mt genome. Mitochondrial genome sequences of 17 species from 12 families were used to reconstruct phylogenetic relationships within the order Pempheriformes. The phylogenetic trees were constructed with three methods (neighbor joining [NJ], maximum likelihood [ML], and Bayesian method) using 12 protein coding genes, but not *ND6*. In all phylogenetic trees, Pempheriformes were clustered into three strongly supported clades. Two Acropomatidae species (*Synagrops japonicus* in clade-Ⅰ and *Doederleinia berycoides* in clade-Ⅲ) were polyphyletic; *S*. *japonicus* was close to Lateolabracidae and was the sister of Glaucosomatidae + (Pempheridae/(Percophidae+Creediidae)), and *D*. *berycoides* was sister to Howellidae + Epigonidae. All phylogenetic trees supported a sister relationship between Creediidae and Percophidae in clade-Ⅰ. Glaucosomatidae formed a sister clade with Pempheridae. The relationships within clade-Ⅱ, which was composed of four families (Pentacerotidae, Polyprionidae, Banjosidae, and Bathyclupeidae), slightly differed between NJ/ML and BI tree topologies. In clade-Ⅲ, the relationships among Howellidae, Epigonidae, and Acropomatidae were strongly supported.

The striped jewfish, *Stereolepis doederleini* Lindberg and Krasyukova, 1969, belongs to the family Polyprionidae (order Pempheriformes), which is composed of two genera and six species worldwide. The genus *Stereolepis* has only two species, *S*. *doederleini* and *S. gigas* Ayres, 1859 (FishBase ver. 12/2019). The striped jewfish, *S. doederleini*, a deep-water demersal fish that inhabits depths of 400-600 m, has been reported from South Korea, Japan, and Russia. In South Korea, *S. doederleini* is commonly called the legendary fish and is highly priced.

The phylogeny of Pempheriformes has been controversial. Betancur-R *et al*. (2017) established the order Pempheriformes to include 14 families previously classified by Nelson *et al*. (2016) within the orders Trachiniformes and Perciformes. Thus, Betancur-R *et al.* (2017) transferred Champsodontidae, Creediidae, Leptoscopidae, and Percophidae from Trachiniformes and Acropomatidae, Banjosidae, Bathyclupeidae, Epigonidae, Glaucosomatidae, Howellidae, Lateolabracidae, Pempheridae, Pentacerotidae, and Polyprionidae from Perciformes. They added Hemerocoetidae, Ostracoberycidae, and Symphysanodontidae to their new order Pempheriformes. Betancur-R *et al.* (2017) placed Dinolestidae and Scombropidae, two families previously treated within Perciformes by Nelson *et al*. (2016), within their Eupercaria (*incertae sedis*) and Scombriformes, respectively. Ghedotti *et al*. (2018) proposed the transfer of Dinolestidae, Scombropidae, and all the families in Pempheriformes *sensu* Betancur-R *et al*. (2017), excluding Percophidae, to Acropomatiformes and further expanded the order to include two new families, Malakichthyidae and Synagropidae.

Here, we sequenced the complete mitochondrial (mt) genome of the striped jewfish, *S. doederleini*, which, to the best of our knowledge, is the first time an mt genome has been sequence for a fish in the family Polyprionidae. We also conducted phylogenetic analysis to examine relationships within the Pempheriformes.

The genomic DNA was extracted using a NucleoSpin Tissue kit (Macherey-Nagel, Germany) from fin tissue of *S. doederleini* obtained from a commercial aquarium in Jeju Island. The complete mt genome was amplified using eight pairs of degenerate primers, and several internal primers were used for primer walking sequencing. The complete mt genome was annotated using the web-based tool MITOS (Bernt *et al*., 2013), followed by manual validation of the coding regions, using the NCBI ORF Finder, and by comparison with other known mt genomes of Pempheriformes. Graphical genome maps were generated using the SnapGene 5 software.

To reconstruct phylogenetic relationships within the order, we downloaded 16 complete mt genome sequences of Pempheriformes from the GenBank database (Table 1) and used sequences of 12 protein-coding genes (PCGs); sequences of NADH dehydrogenase subunit 6 gene were not included in the analysis because of their heterogeneous base composition and consistently poor phylogenetic performance (Zardoya and Meyer, 1996; Miya and Nishida, 2000). The MEGA X software (Kumar *et al*., 2018), for sequence alignment, neighbor joining (NJ), and maximum likelihood (ML), and MrBayes 3.2 software (Ronquist *et al*. 2012), for Bayesian inference (BI), were used for phylogenetic tree reconstruction. GTR+G+I model was selected as the best evolutionary model for ML and BI by jmodeltest2 (Darriba *et al*., 2012). Bootstrap analysis (1,000 replicates) was conducted for ML tree reconstruction and four Markov chains for BI were run for 3,000,000 generations and sampled every 100 generations to obtain a posterior probability (PP) distribution of 1,000 trees. *Macquaria ambigua* (Centrarchiformes, Percichthyidae) was used as an outgroup.

**Table 1 -:** List of species used for phylogenetic analysis.

Order	Family	Scientific name	GenBank
Pempheriformes	Acropomatidae	*Doederleinia berycoides*	AP009181
		*Synagrops japonicus*	AP017439
	Banjosidae	*Banjos banjos*	KT345965
	Bathyclupeidae	*Bathyclupea megaceps*	AP017448
	Creediidae	*Limnichthys fasciatus*	AP017453
	Epigonidae	*Epigonus denticulatus*	AP017435
	Glaucosomatidae	*Glaucosoma buergeri*	AP018347
	Howellidae	*Howella brodiei*	AP014536
	Lateolabracidae	*Lateolabrax japonicus*	AP006789
		*Lateolabrax latus*	KR780681
	Pempheridae	*Pempheris schwenkii*	AB355922
	Pentacerotidae	*Pseudopentaceros wheeleri*	AB741956
		*Histiopterus typus*	AP006807
		*Pentaceros japonicus*	AB739063
		*Pseudopentaceros richardsoni*	AB734122
	Percophidae	*Acanthaphritis unoorum*	AP017452
	Polyprionidae	*Stereolepis doederleini*	This study
Centrarchiformes	Percichthyidae	*Macquaria ambigua*	AP014533

The complete mt genome of *S. doederleini* was 16,513 bp long (GenBank accession number MT083886) and showed general features (13 PCGs, 22 tRNAs, 2 rRNAs, and control region) and gene order typical for bony fishes (Figure 1).

**Figure 1 -:**
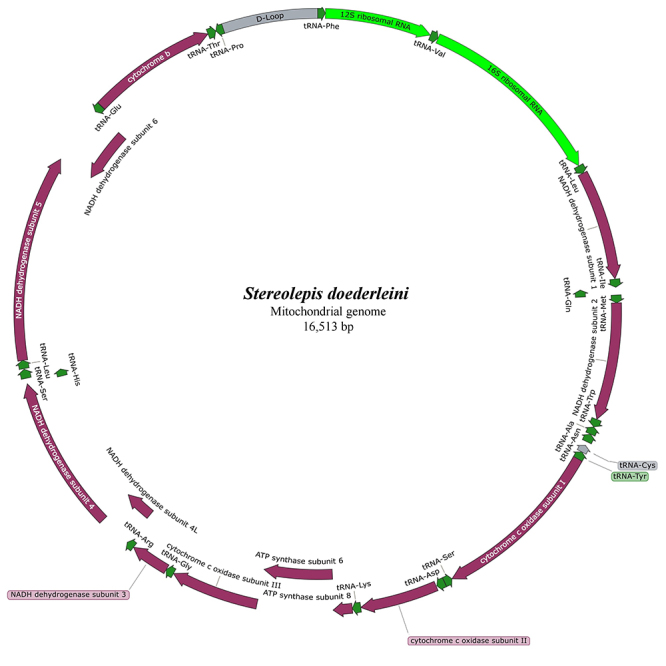
Mitochondrial genome of *Stereolepis doederleini*. The genes and their order was identical to those of other bony fishes.

Similar to other bony fishes, ATG is the start codon of all PCGs in the *S. doederleini* mt genome, with the exception of cytochrome *c* oxidase subunit I (COI), in which ATG is replaced with GTG (Oh and Jung, 2008; Zhong *et al*., 2018). ATG is the most common initiation codon for 13 PCGs in sequenced vertebrate mt genomes, although there are exceptions in which the start codons were ATA, ATC, ATT, GTG, and GTT (Miya and Nishida, 1999; Miya *et al*., 2003). Most PCGs of *S. doederleini* mt genome ended with TAA, except for COII and cytochrome *b* (incomplete T), and NADH dehydrogenase subunit 4 (AGA). The alternative codon and incomplete termination is often found in various bony fishes (Miya *et al*., 2003; Zhong *et al*., 2018; Li *et al*., 2019). 

The tree topologies obtained by NJ, ML, and BI were congruent in resolving three clades within Pempheriformes (Figure 2). Clade-I comprised Creediidae, Percophidae, Pempheridae, Glaucosomatidae, Acropomatidae-I, and Lateolabracidae; Clade-II was composed of Pentacerotidae, Polyprionidae, Banjosidae, and Bathyclupeidae; and Clade-III included Howellidae, Epigonidae, and Acropomatidae-II. Acropomatidae was polyphyletic (*S. japonicus* was placed within Clade-I and *D. berycoides* within Clade-III) and *Pseudopentaceros* in Clade-II was paraphyletic (*Pentaceros* was within the clade with the two *Pseudopentaceros* species). Acropomatidae-I was closely related to Lateolabracidae and Glaucosomatidae, and Acropomatidae-II was sister to Howellidae + Epigonidae.

**Figure 2 -:**
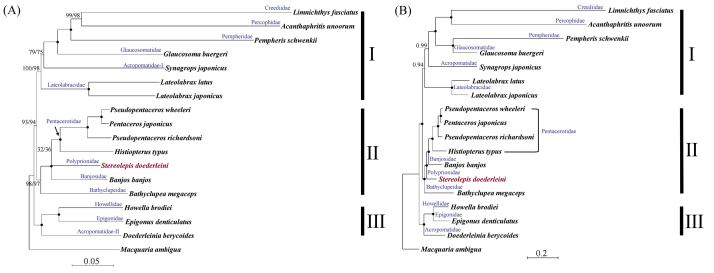
Phylogeny of Pempheriformes using 12 protein-coding genes (excluding *ND6*) of the mitochondrial genome. (A) Neighbor-joining (NJ), maximum likelihood (ML), and (B) Bayesian inference (BI) showed that the values on the nodes represent bootstrap support and posterior probability (100% and 1.0 support value is shown as a black circle on each node, respectively). *Macquaria ambigua* (Centrarchiformes, Percichthyidae) was used as an outgroup.

Although NJ and ML tree suggested that a common ancestor diverged into Polyprionidae and Pentacerotidae with low support (Figure 2A), the branch of *S. doederleini* was diverged earlier than Banjosidae and Pentacerotidae with strongly support in BI (Figure 2B).

The phylogenetic analysis resolved Acropomatidae polyphyletic and *Pseudopentaceros* paraphyletic (Sanciangco *et al*., 2016; Niu *et al*., 2018). *Synagrops* (Acropomatidae) is currently considered an unnatural group characterized by several anatomical traits such as the absence of pelvic-fin spine serrations (Schwarzhans and Prokofiev, 2017). Therefore, the taxonomy of Acropomatidae, including that of *D. berycoides*, as well as that of *Pentaceros* and *Pseudopentaceros* should be revised to reflect the phylogenetic status of the groups. Percophidae had been classified within Perciformes (Van Der Laan *et al*., 2014; Mirande, 2017). However, our data and other studies placed Percophidae within Pempheriformes (Sanciangco *et al*., 2016; Satoh, 2018).

Among the NJ, ML, and BI trees, the topology of the BI tree was most similar to that reported by Betancur-R *et al.* (2017), although the phylogenetic relationships indicated in these two trees were slightly different (Figure 3). Betancur-R *et al.* (2017) suggested that Acropomatidae was close to Lateolabracidae in Clade-I, and that Acropomatidae was close to Howellidae/Ostracoberycidae in Clade-III. However, the BI tree constructed in the present study indicated that Acropomatidae-I was not directly grouped with Lateolabracidae in Clade-I, whereas we found that Epigonidae was grouped with Howellidae in Clade-III. Although the support for Pempheriformes was low due to a lack of morphological synapomorphies (Betancur-R *et al.,* 2017), our molecular data strongly supported most of phylogenetic relationships among Pempheriformes reported by Betancur-R *et al.* (2017; Figure 3).

**Figure 3 -:**
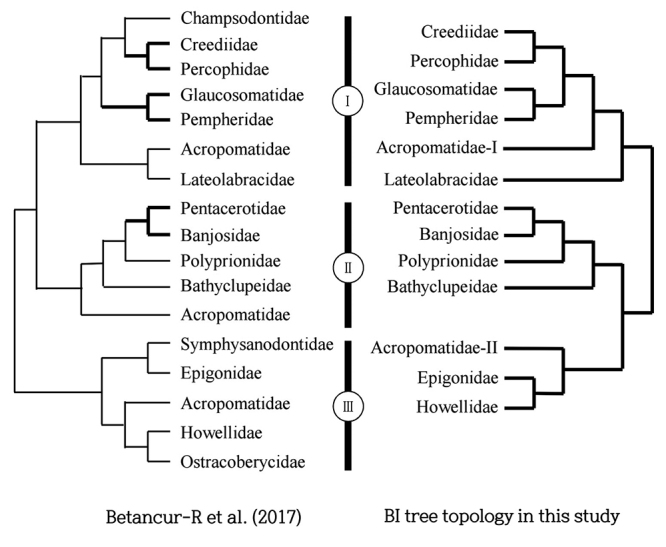
Comparison of the topologies of the present and previous classifications. (Left) Edited topology of the classification presented by Betancur-R *et al*. (2017). (Right) Topology of the BI tree constructed in the present study. Bold lines indicate the highly reliable branches (support value > 50% in left and support value > 90% in right).

In this study, we included sequences of 12 of the 20 families in the order Pempheriformes. For a complete resolution of the phylogenetic relationships between families within Pempheriformes, future phylogenetic studies should include representatives of the remaining eight families.

## References

[B1] Bernt M, Donath A, Jühling F, Externbrink F, Florentz C, Fritzsch G, Pütz J, Middendorf M, Stadler PF (2013). MITOS: improved de novo metazoan mitochondrial genome annotation. Mol Phylogenet Evol.

[B2] Betancur-R R, Wiley EO, Arratia G, Acero A, Bailly N, Miya M, Lecointre G, Orti G (2017). Phylogenetic classification of bony fishes. BMC Evol Biol.

[B3] Darriba D, Taboada GL, Doallo R, Posada D (2012). jModelTest 2: more models, new heuristics and parallel computing. Nat Methods.

[B4] Ghedotti MJ, Gruber JN, Barton RW, Davis MP, Smith WL (2018). Morphology and evolution of bioluminescent organs in the glowbellies (Percomorpha: Acropomatidae) with comments on the taxonomy and phylogeny of Acropomatiformes. J Morphol.

[B5] Kumar S, Stecher G, Li M, Knyaz C, Tamura K (2018). MEGA X: molecular evolutionary genetics analysis across computing platforms. Mol Biol Evol.

[B6] Li R, Wang G, Wen ZY, Zou YC, Qin CJ, Luo Y, Wang J, Chen GH (2019). Complete mitochondrial genome of a kind of snakehead fish Channa siamensis and its phylogenetic consideration. Genes Genom.

[B7] Mirande JM (2017). Combined phylogeny of ray‐finned fishes (Actinopterygii) and the use of morphological characters in large‐scale analyses. Cladistics.

[B8] Miya M, Nishida M (1999). Organization of the mitochondrial genome of a deep-sea fish, Gonostoma gracile (Teleostei: Stomiiformes): first example of transfer RNA gene rearrangement in bony fishes. Mar Biotechnol.

[B9] Miya M, Nishida M (2000). Use of mitogenome information in teleostean molecular phylogenetics: a tree-based exploration under the maximum-parsimony optimality criterion. Mol Phylogenet Evol.

[B10] Miya M, Takeshima H, Endo H, Ishiguro NB, Inoue JG, Mukai T, Satoh TP, Yamaguchi M, Kawaguchi A, Mabuchi K (2003). Major patterns of higher teleostean phylogenies; a new perspective based on 100 complete mitochondrial DNA sequences. Mol Phylogenet Evol.

[B11] Nelson JS, Grande T, Wilson MVH (2016). Fishes of the World.

[B12] Niu W, Kong L, Ma H, Gao Y (2018). Characterization and phylogenetic analysis of the complete mitochondrial genome of Bodianus oxycephalus (Perciformes, Labridae). Conserv Genet Resour.

[B13] Oh DJ, Jung YH (2008). The mitochondrial genome of the threespot wrasse Halichoeres trimaculatus (Perciformes, Labridae). Genes Genom.

[B14] Ronquist F, Teslenko M, van der Mark P, Ayres DL, Darling A, Hohna S, Larget B, Liu L, Suchard MA, Huelsenbeck JP (2012). MrBayes 3.2: efficient Bayesian phylogenetic inference and model choice across a large model space. Syst Biol.

[B15] Sanciangco MD, Carpenter KE, Betancur-R R (2016). Phylogenetic placement of enigmatic percomorph families (Teleostei: Percomorphaceae). Mol Phylogenet Evol.

[B16] Satoh TP (2018). Complete mitochondrial genome sequence of Glaucosoma buergeri (Pempheriformes: Glaucosomatidae) with implications based on the phylogenetic position. Mitochondr DNA Part B.

[B17] Schwarzhans WW, Prokofiev AM (2017). Reappraisal of Synagrops Günther, 1887 with rehabilitation and revision of Parascombrops Alcock, 1889 including description of seven new species and two new genera (Perciformes: Acropomatidae). Zootaxa.

[B18] Van Der Laan R, Eschmeyer WN, Fricke R (2014). Family-group names of recent fishes. Zootaxa.

[B19] Zardoya R, Meyer A (1996). Phylogenetic performance of mitochondrial protein-coding genes in resolving relationships among vertebrates. Mol Biol Evol.

[B20] Zhong L, Wang M, Li D, Tang S, Zhang T, Bian W, Chen X (2018). Complete mitochondrial genome of freshwater goby Rhinogobius cliffordpopei (Perciformes, Gobiidae): genome characterization and phylogenetic analysis. Genes Genom.

